# Supplementary material for: End Sequence Analysis Toolkit (ESAT) expands the extractable information from single-cell RNA-seq data
Source: Genome Res. 2016 Oct;26(10):1397–410. doi: 10.1101/gr.207902.116 (PMC5052061; doi:10.1101/gr.207902.116)
Supplement: Supplemental Material [file supp_gr.207902.116_Supp_Materials.tar.gz › ESAT-esat_v0.1/doc/html/functions_func_l.html]

ESAT: Class Members - Functions


ESAT


- Main Page
- Related Pages
- Packages
- Classes
- Files

- Class List
- Class Index
- Class Hierarchy
- Class Members

- All
- Functions
- Variables
- Enumerator

- a
- b
- c
- d
- e
- f
- g
- h
- i
- j
- k
- l
- m
- n
- o
- p
- q
- r
- s
- t
- u
- v
- w
- z


All Classes Namespaces Files Functions Variables Enumerator Pages

### - l -

- l2a()
  : umms.core.scripture.BuildScriptureCoordinateSpace
- lastIdxWhereListSearchBroke()
  : broad.core.annotation.BasicGenomicAnnotation
- leftIntersect()
  : broad.core.math.EmpiricalDistribution
- length()
  : broad.core.annotation.BasicLightweightAnnotation
  , broad.core.annotation.LightweightGenomicAnnotation
  , broad.core.multiplealignment.MAFAlignment
  , broad.core.multiplealignment.MultipleAlignment
  , broad.core.sequence.SequenceRegion
  , broad.pda.datastructures.Alignments
  , broad.pda.feature.genome.Chromosome
  , jaligner.Sequence
  , umms.core.annotation.Annotation
  , umms.core.annotation.BasicAnnotation
  , umms.core.annotation.CompoundInterval
- LengthScore()
  : umms.core.model.score.LengthScore
- listFromArray()
  : broad.core.util.CLUtil
- load()
  : broad.core.annotation.AnnotationReader< T extends GenomicAnnotation >
  , broad.core.annotation.BasicGenomicAnnotationReader
  , broad.core.annotation.BEDGraphReader
  , broad.core.annotation.BEDReader
  , broad.core.annotation.RepeatMaskerAnnotationReader
  , broad.core.annotation.ShortBEDReader
  , broad.core.motif.PositionWeightMatrixIO
  , broad.core.multiplealignment.FastaMultipleAlignmentIO
  , broad.core.multiplealignment.MAFAlignment
  , broad.core.multiplealignment.MAFIO
  , broad.core.multiplealignment.MultipleAlignmentIO
  , broad.core.multiplealignment.PhylipInterleavedMultipleAlignmentIO
  , broad.core.multiplealignment.PhylipSequencialMultipleAlignmentIO
  , broad.core.siphy.StationaryDistributionIO
  , broad.core.siphy.TreeScalerIO
  , broad.pda.datastructures.AlignmentsReader
  , broad.pda.seq.alignment.StoredAlignmentStats
  , umms.core.annotation.AnnotationFileReader
- loadAlignmentData()
  : broad.pda.annotation.BEDFileParser
  , broad.pda.seq.alignment.AlignmentUtils
- loadAlignmentDataByChr()
  : broad.pda.annotation.BEDFileParser
- loadAlignmentDataByChr3Prime()
  : broad.pda.annotation.BEDFileParser
- loadAlignmentDataByChr5Prime()
  : broad.pda.annotation.BEDFileParser
- loadAlignmentDataByChrArrayList()
  : broad.pda.annotation.BEDFileParser
- loadAlignmentDataToTree()
  : broad.pda.annotation.BEDFileParser
- loadAlignmentModel()
  : broad.pda.seq.rap.GenomeCommandLineProgram
  , broad.pda.seq.rap.rna.TranscriptomeCommandLineProgram
- loadAll()
  : broad.core.sequence.FastaSequenceIO
- loadAnnotations()
  : umms.core.utils.AnnotationUtils
- loadAtMostRandom()
  : broad.core.siphy.TreeScalerIO
- loadChrSizes()
  : broad.pda.annotation.BEDFileParser
- loadCoordinateSpace()
  : broad.pda.seq.rap.GenomeCommandLineProgram
  , broad.pda.seq.rap.PlotAggregateRegions
  , broad.pda.seq.rap.rna.TranscriptomeCommandLineProgram
- loadData()
  : broad.core.util.ParseGCTFile
  , broad.pda.annotation.BEDFileParser
- loadDataByChr()
  : broad.pda.annotation.BEDFileParser
- loadDataByChrToTree()
  : broad.pda.annotation.BEDFileParser
- loadDataByName()
  : broad.pda.annotation.BEDFileParser
- loadDataLine()
  : broad.pda.annotation.BEDFileParser
- loadedPairsAsFragments()
  : broad.pda.seq.segmentation.GenericAlignmentDataModel
- loadFromAscii()
  : broad.core.sequence.FastaQualityIndex
- loadGoMap()
  : broad.pda.gene.functionalAnnotation
- loadGTF()
  : broad.pda.annotation.BEDFileParser
- loadIndex()
  : broad.core.multiplealignment.MAFAlignment
- loadIntegrated()
  : broad.core.siphy.TreeScalerIO
- loadIntoDistribution()
  : broad.core.siphy.StationaryDistributionIO
  , broad.core.siphy.TreeScalerIO
- loadIsoformDataByChrToTree()
  : broad.pda.annotation.BEDFileParser
  , broad.pda.annotation.GTFFileParser
- loadList()
  : broad.pda.annotation.BEDFileParser
- loadOldIntegrated()
  : broad.core.siphy.TreeScalerIO
- loadPIDName()
  : broad.core.util.ParseGCTFile
- loadRNAAlignmentModel()
  : broad.pda.seq.rap.rna.TranscriptomeCommandLineProgram
- loadSequence()
  : broad.pda.feature.genome.Chromosome
- loadSequences()
  : broad.core.sequence.FastaSequenceIO
- loadSequencesByNameFromDirectory()
  : broad.core.sequence.FastaSequenceIO
- LocationAwareMatrix()
  : broad.pda.datastructures.LocationAwareMatrix
- Locus()
  : broad.pda.annotation.Locus
- Loess()
  : broad.core.math.Loess
- log10()
  : broad.core.datastructures.MatrixWithHeaders
- log2()
  : broad.core.datastructures.MatrixWithHeaders
  , broad.core.math.Loess
  , broad.core.math.MathUtil
  , broad.core.util.CLUtil
- logTransform()
  : broad.core.util.ParseGCTFile
- lu()
  : Jama.Matrix
- LUDecomposition()
  : Jama.LUDecomposition


---

Generated on Fri Aug 15 2014 15:11:16 for ESAT by  

 1.8.7
